# A253 IMPACT OF THE COVID-19 PANDEMIC LOCKDOWN ON PHYSICAL ACTIVITY LEVELS IN CHILDREN AND ADOLESCENTS WITH INFLAMMATORY BOWEL DISEASE IN QUEBEC

**DOI:** 10.1093/jcag/gwab049.252

**Published:** 2022-02-21

**Authors:** J Bouthot, L Dehbidi Assadzadeh, L Belmesk, S Madagh, S Geng, C Deslandres, P Jantchou

**Affiliations:** 1 Universite de Montreal, Montreal, QC, Canada; 2 Service de gastro-entérologie, CHU Sainte-Justine, Montréal, QC, Canada

## Abstract

**Background:**

An average daily moderate-to-vigorous physical activity (MVPA) of at least 60 minutes was recommended by the Canadian Guidelines for children. Because of lockdown restrictions during the COVID-19 pandemic, maintaining physical activity levels (PAL) has been a challenge for youth.

**Aims:**

The primary aim of this study was to compare MVPA levels in children with inflammatory bowel diseases (IBD) before and after this period. The secondary aims were to assess clinical factors that might influence any changes in MVPA patterns.

**Methods:**

Patients with IBD, age ≥5 years, were enrolled in a prospective study on PAL starting June 2018 (self-reported questionnaires during outpatient visits). They were then surveyed online at the end of the second lockdown in July-August 2021. PAL were assessed with the Canadian Health Measure Survey Children-Physical Activity Questionnaire. The responses were converted into metabolic equivalents of tasks by using validated tables. Influence of clinical factors of IBD on changes in MVPA was assessed. A multivariate logistic regression was performed to investigate the association between several risk factors and PAL.

**Results:**

We included 72 patients (38 males; mean (SD) age 17.0 (2.89) years, 48 (66.7%) diagnosed with Crohn’s disease, 19 (26.4%) with ulcerative colitis, and 5 (6.9%) with indeterminate colitis). At last follow-up, 90.3% were in clinical remission according to validated disease activity score. During summer 2021, 16.7% of patients reached the Canadian PAL target, compared to 38.9% before the pandemic. The median daily duration of MVPA in summer 2021 decreased from 37 (Interquartile range (IQR) = 3–82) to 21 (IQR=3–40) minutes. The proportion of sedentary patients increased by 1.4% (37.5% to 38.9%). The proportion of extremely active patients decreased by 16.7% (27.8% to 11.1%), while moderately active patients increased by 20.9% (8.3% to 29.2%). Age, gender, disease type and activity were not significantly associated with the PAL at baseline or at follow-up.

**Conclusions:**

This study found a significant decrease in PAL and time spent doing MVPA in children with IBD in Quebec following the COVID-19 pandemic lockdown. While the recommended target was far from being met before the pandemic, the gap has widened further during the pandemic. We found no clinical factors associated with the PAL. The impact of low PAL on well-being, weight, disease activity and quality of life will be assessed during the follow-up of this cohort.

**Funding Agencies:**

None

